# Surface modification of small intestine submucosa in tissue engineering

**DOI:** 10.1093/rb/rbaa014

**Published:** 2020-05-18

**Authors:** Pan Zhao, Xiang Li, Qin Fang, Fanglin Wang, Qiang Ao, Xiaohong Wang, Xiaohong Tian, Hao Tong, Shuling Bai, Jun Fan

**Affiliations:** r1 Department of Tissue Engineering, School of Fundamental Sciences, China Medical University, 77 Puhe Avenue, Shenbei New District, Shenyang 110122, China; r2 Department of Cell Biology, School of Life Sciences, China Medical University, 77 Puhe Avenue, Shenbei New District, Shenyang 110122, China; r3 Cardiac Surgery, Liaoning First Hospital of China Medical University, No. 155 Nanjing Street, Heping District, Shenyang, Liaoning 110122, China

**Keywords:** small intestinal submucosa, surface modification, *in vitro* scaffold, tissue engineering

## Abstract

With the development of tissue engineering, the required biomaterials need to have the ability to promote cell adhesion and proliferation *in vitro* and *in vivo*. Especially, surface modification of the scaffold material has a great influence on biocompatibility and functionality of materials. The small intestine submucosa (SIS) is an extracellular matrix isolated from the submucosal layer of porcine jejunum, which has good tissue mechanical properties and regenerative activity, and is suitable for cell adhesion, proliferation and differentiation. In recent years, SIS is widely used in different areas of tissue reconstruction, such as blood vessels, bone, cartilage, bladder and ureter, etc. This paper discusses the main methods for surface modification of SIS to improve and optimize the performance of SIS bioscaffolds, including functional group bonding, protein adsorption, mineral coating, topography and formatting modification and drug combination. In addition, the reasonable combination of these methods also offers great improvement on SIS surface modification. This article makes a shallow review of the surface modification of SIS and its application in tissue engineering.

## Introduction

Tissue engineering is a discipline that combines materials science with cell biology and is dedicated to the repair and reconstruction of tissues *in vitro* and *in vivo*. Scientists use cells and cytokines, physical and chemical factors to maintain and repair the damaged tissue. Seed cells, scaffold materials and cytokines are the main elements of tissue engineering. Scaffold biomaterials are not only used for physical support but also can extensively influence the cell fate by cell–surface interactions [1, 2]. The material stimuli will convert into biochemical signals in cells. Physicochemical properties of the biomaterial greatly influence cell adhesion, proliferation and differentiation on the scaffold [3, 4]. Cells in contact with scaffold materials will feel their properties and guide transcription factors those regulate cell fate and differentiation. For example, Kim *et al*. reported that the fibrinogen coating (biphasic calcium phosphate, BCP) on the surface of the material significantly changed the adhesion and proliferation of human mesenchymal stem cells. The process is affected by changes of the surface roughness due to the function of the β15-42 epitope region contained in fibrinogen [5].

Scaffolds are produced from natural and synthetic biomaterials. The synthetic materials have polymer properties that are easy to control but lack the biochemical activity compared with the natural materials. Many biological materials, such as collagen, can provide the extracellular matrix (ECM) components required for cell adhesion, thereby accelerating cell growth and function. For synthetic polymeric materials, although cells can adhere, they require additional energy to generate ECM [6]. The natural biomaterials have good biochemical activity and biological compatibility. However, in the process of handling natural materials, such as the decellularized tissues, the normal 3D structure is changed or some biological factors are lost [7]. Furthermore, the requirements of material characteristics vary with different kinds of tissue construction. The surface modification is to change the biological activity of the cells on the surface of the material without changing the integrity and physical properties of the overall skeleton of the material [8]. Therefore, the surface modification treatment of natural materials is necessary and very important in tissue engineering [9].

Small intestine submucosa (SIS) is an excellent bioscaffold. Our experimental results and numerous studies have shown that SIS has great prospects for allogeneic transplantation and damage repair [10, 11]. SIS is a natural ECM material with a 3D structure. About 40% of its dry mass is collagen tissue. SIS is mainly composed of type I and type III collagen, and a small amount of IV, V and type II collagen, in addition to aminoglucan and glycoprotein [12]. The interdependence of various matrix components of SIS is closely related to maintain the fine structure and microenvironment of the tissue, not only as a scaffold but also with special physiological functions. Collagen exists in the form of collagen fibers in the body, and its fibrous structure is extremely advantageous for adhesion, growth and proliferation of cells. Fibronectin in SIS is second only to collagen, and its ligand plays an adhesive role between cells and matrix, cells and cells. It is often used as a substrate for *in vitro* cell culture and is used to coat modified synthetic scaffolds. SIS contains a variety of cytokines such as transforming growth factor-beta, vascular endothelial growth factor (VEGF), basic fibroblast growth factor, epidermal growth factor and insulin-like growth factor-1, fibronectin, heparin, glycosaminoglycan, chondroitin sulfate, hyaluronic acid (HA) and heparin sulfate, etc. [13]. SIS provides the host cell with a natural environment of attachment and migration, increases its biocompatibility with the host cell and can quickly bind to the host tissue, promote angiogenesis and restore tissue function.

These above characteristics of SIS show significant advantages in regenerative medicine and tissue engineering. Therefore, SIS has been widely used in the field of tissue engineering on repairing damaged tissues and organs, including angiocarpy [14–16], bone and cartilage [17], tympanic membrane [18], meniscus [19], ligament [20], vocal cord [21, 22], urinary system [23], skin [24], etc.

However, there are still some shortcomings in SIS. Such as, the surface structure is different from the ECM, which causes the scaffold to be inadequate in anticoagulation and cell adhesion [25]. The lower mechanical strength of SIS also limited its application in tissue engineering [26]. On this basis, a series of modification of SIS was carried out to meet actual needs.

For biological materials, there are usually two types of modification methods, batch and surface modification. Batch modification will make relatively large adjustments to the entire biological material, even devices that are not exposed to the outside of the cell, so a comprehensive assessment of the material is needed [8]. In contrast, the functions added by surface modification are concentrated on the surface of the material. As the surface begins to degrade, the functionality is gradually lost, which is beneficial in tissue engineering and regenerative medicine [27]. In the early stage of surface modification, cells need the help and support of foreign functions. Once the tissue function reaches relative maturity, the cells will tend to mature cytokines and exercise their functional mission [28]. Therefore, the surface modification will not change the basic material, and the change of biological activity will not be sacrificed too much. Therefore, surface modification is particularly effective for the improvement of biological scaffolds.

In this review, we discuss five basic and commonly used methods of surface modification of scaffold SIS, including functional group bonding, protein adsorption, mineral coating, topological modification and drug combination, and established their development process, purpose and unique characteristics [29–33]. Each method has a unique contribution to surface modification. The studies have proven that the above methods can promote the formation of the cellular microenvironment on the surface of SIS. These methods introduced in the review are to provide some ideas and a comprehensive perspective for future studies.

## Overview of SIS

SIS has a pale white appearance and a thickness of about 80–100 µm. SIS can be divided into three layers structurally, mucosal layer, serous layer and muscular layer. The mucosal side is smooth and the muscle layer is relatively rough. After lyophilization, SIS appears translucent. The collagen fibers in the SIS present a 3D network-like structure. Under scanning electron microscope (SEM) observation, the mucosal and muscular layers are composed of collagen fiber bundles intertwined into a network. The muscle layer has thick collagen fibers with a loose structure, and the mucosal surface collagen fibers are thin and relatively dense.

### Preparation

**Figure 1.**
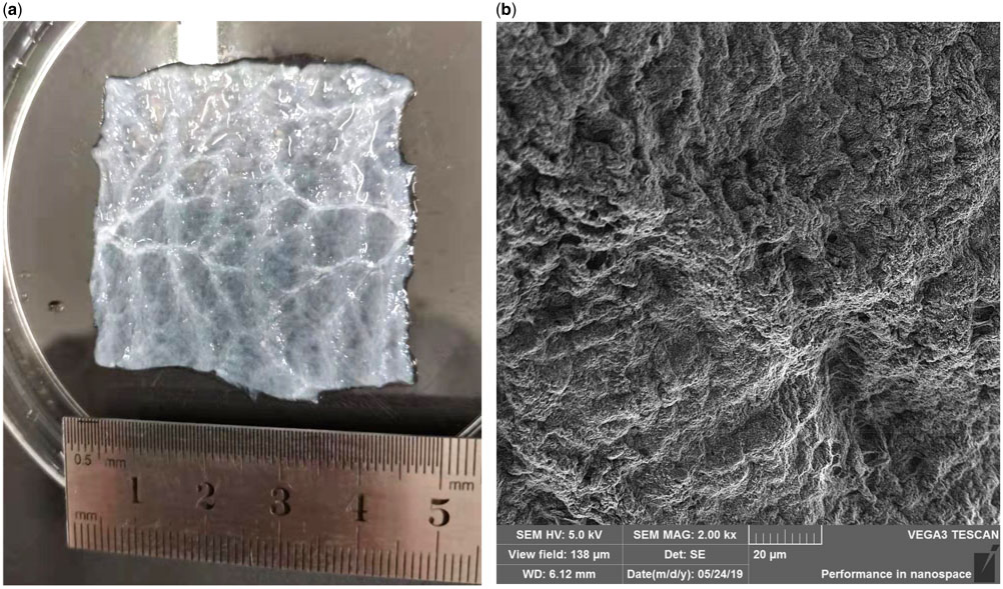
(**a**) The macroscopic surface morphology of the SIS after decellularization and (**b**) the SEM image of the SIS after decellularization.

Preparation method of SIS has a long history. It is mainly divided into physical method and chemical method. The physical method is to physically remove the mucosal layer, the serosal layer and the muscular layer of the small intestine of the pig, and then separate the submucosal layer of the small intestine. The main purpose of the chemical method is to remove cells from the tissue and avoid immune rejection after transplantation. The chemical method is to immerse the tissue after the physical method in a series of solutions. First, the tissue was soaked in a solution containing 100 mM Ethylene Diamine Tetraacetic Acid (EDTA) and 10 mM NaOH (PH = 11–12) for 16 h, then in a solution of 1 M NaCl and 1 M HCl (PH = 1) for 6–8 h, and then in a phosphate-buffered saline (PBS) solution containing 1 M NaCl (PH PBS = 7–7.4) for 16 h and finally incubate in PBS (PH = 7–7.4) for 2 h. After rinsing with deionized water, immerse in 20% ethanol of 0.1% peroxyacetic acid for 8 h, rinse with sterile water for 2 h and then store at −80°C. Figure 1 shows the macroscopic surface morphology of the SIS after decellularization (Fig. 1a) and the microscopic surface morphology of the SEM image (Fig. 1b).

Decellularized biomaterials have no immunological rejection and become an ideal material for biological scaffolds [34]. Histological analysis showed that physical and chemical treatment completely removed the cells contained in SIS itself, and collagen fibers appeared in turn. It retained the normal 3D structure of SIS, rich grids and continuous fibers, so it could promote cell adhesion to a certain extent proliferation. Enzyme-linked immuno sorbent assay (ELISA) showed that various growth factor contents were obtained. As for the biological activities of various growth factors, although no researchers have specifically quantified them, many experimenters have implanted pure SIS into various animals and found that it promotes tissue regeneration and healing, including the uterus and dural defect repair [35–38], which indirectly illustrates the existence of growth factor activity. This also provides a certain basis for future quantitative activity detection. CCK-8 also shows that SIS scaffolds are nontoxic to cells, and various mechanical tests have also shown good mechanical properties [39, 40]. Therefore, SIS is nonimmunogenic and has excellent biocompatibility. It is suitable as an allogeneic scaffold for repairing tissue defects without causing immune rejection [24, 41]. SIS plays an important role in the repair and reconstruction of tissues [42, 43] and is an excellent natural ECM-derived material [44].

To a certain extent, SIS is widely used due to its controllability of shape, excellent fluidity and easy surface modification. SIS has a variety of manifestations depending on the handling process, including sheet, spongy and hydrogels [41].

### Forms of SIS

#### Sheet

**Figure 2.**
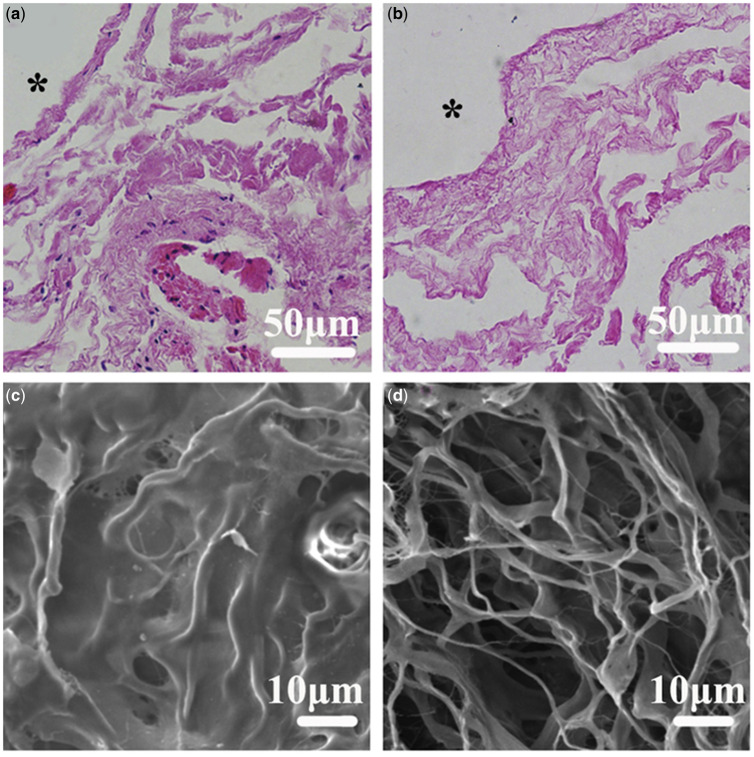
(**a**) HE image of SIS without decellularization, showing many blue nuclei; (**b**) HE image of decellularized SIS, almost no nuclear signs; (**c**) SEM image of undecellularized SIS showed coarse, dense and nonporous; (**d**) SEM image of decellularized SIS shows loose 3D network structure. Reproduced with permission from Fang *et al*. [45].

This is the simplest of the three forms of SIS, and the SIS is decellularized by the physical and chemical steps to obtain a sheet-like SIS. The flaky SIS is white translucent with a thickness of about 0.1 mm. The surface has some fiber lines. Under the SEM, a loose 3D network structure is visible, which is suitable as a scaffold material [45]. Figure 2 shows Hematoxylin-Eosin staining (HE) images and SEM images before and after SIS decellularization.

The flaky SIS can be directly crimped into a small tubular structure and transplanted directly into the recipient as a scaffold material or biological dressing. In recent years, flaky SIS has been widely used in the study of tissue defect repair *in vitro*. The flaky SIS was first used in the study of repairing blood vessels, and vascular endothelial cells were planted on SIS to construct regenerative tissue engineering blood vessels [45, 46]. In addition, SIS is used for bladder reconstruction and repairing bone, cartilage and tendon and other aspects [18, 21, 47].

#### Sponge

The sheet SIS is broken up to obtain SIS powder, mixed with a solution containing acetic acid and pepsin, stirred, freeze-dried and then treated with 1-ethyl-3-(3-dimethylaminopropyl) carbodiimide hydrochloride (EDC). EDC was crosslinked for 24 h, and lyophilized SIS was obtained by freeze-drying to obtain a sponge-inspired SIS [48]. Sponge SIS is both elastic and flexible, easy to handle and has interconnected porous structures. The data show that SIS sponge can play a huge role in wound healing and adhere evenly on the wound surface, and the SIS sponge with porous structure shows better wound exudation absorption than covered with polyurethane film [24]. The wound is covered by a thin layer of skin and fibrous tissue is observed at the wound. It can, therefore, be used for the regeneration of skin tissue in the wound area. Based on this discovery, we can predict that SIS sponge also has great prospects in vascular endothelialization. Some researchers have also inoculated rat bone marrow stem cell on SIS sponges [48], which have also been shown to promote cell proliferation.

#### Hydrogel

In addition to sheet and sponge, another common application form of SIS is hydrogel. Hydrogels are considered to be the most suitable tissue fillers because hydrogels can be arbitrarily formed into different shapes by syringes to accommodate tissue defects and regeneration [49].

**Figure 3.**
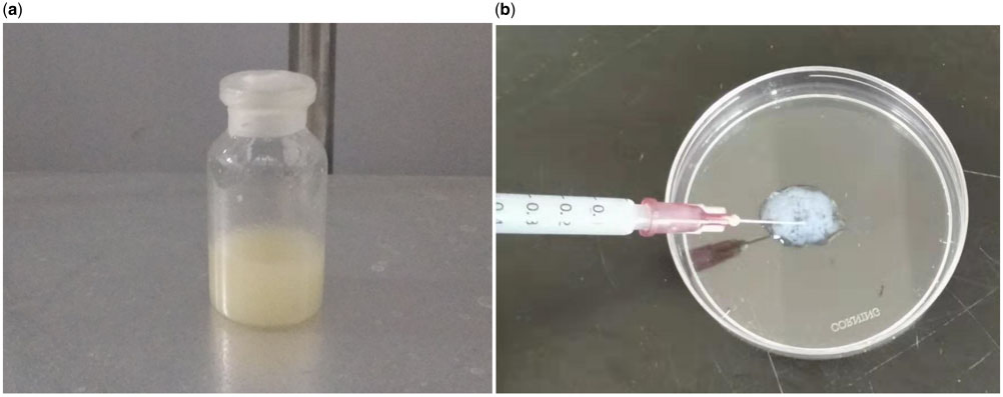
Images of the SIS hydrogel in a vial (**a**) and in a syringe (**b**).

The brief preparation of the hydrogel is as follows. The porcine SIS piece was pulverized, suspended in HCl solution with porcine pepsin, stirred at room temperature, adjusted to pH 7.4, lyophilized and pulverized to obtain an SIS powder. The SIS powder was dissolved in PBS at 20% w/v and shaped into a gel of any shape overnight at 37 °C [41, 47, 50]. For example, SIS hydrogel placed on the bottom of the culture dish forms a flat shape [41, 51], or put in a small bottle forms a thicker cylindrical shape. The hydrogel can also be injected into a specific area with a syringe based on the good fluidity of hydrogel. Figure 3 shows images of the gel in a vial (Fig. 3a) and a syringe (Fig. 3b).

### Application of SIS

As a collagen scaffold, SIS was successfully used for the repair of various tissues. In 1989, Badylak *et al*. applied SIS to replace canine arterial blood vessels [52]. Consequently, they conducted a series of experiments on organs and tissues repair using SIS. They used SIS to replace the superior vena cava of dogs [53], and repair canine achilles tendon [54 and rodent abdominal wall defects [55]. In 1996, SIS was used as a dura mater substitute [56] and bladder repair [57]. SIS was also used as bone repair material in 1999 [58] and repaired ligament healing in 2004 [59].

In the practical applications, some attempts were applied on SIS for enhancing its function. For example, the SIS is made into a tubule with crosslinking of various biologically active factors and transplanted into animal to substitute carotid arteries [23, 47]. SIS was loaded with cardiomyocytes and then was transplanted to the outer surface of infarcted myocardium in mice to repair heart function [16]. For meniscus reconstruction, meniscus cells and synovial-derived stem cells were also transplanted on SIS [19]. Meanwhile, a composite gel composed of SIS and mesenchymal stem cells was created for tissue regeneration [2].

## Surface modification and practical application of SIS

The application of SIS biological scaffolds in tissue engineering is limited by its single biological characteristics and low mechanical properties. Surface modification of SIS hugely improved its application in tissue engineering. Easy surface modification means that the SIS film can be subjected to various forms of surface modification, such as composite cells on the SIS surface, growth factors or high molecular polymers, etc. This will be described in detail below.

### Functional group bonding

Immobilizing biological macromolecules and composite cells on the surface of SIS is the simplest type of surface modification. In order to profoundly change the chemistry of the scaffold, it is usually necessary to have functional groups on the surface of the SIS material. These groups provide richer biological activity for SIS materials. In other words, their specific linkage can crosslink more foreign factors and groups. Methods for functionalizing the surface of materials include plasma deposition, physical embedding of functional molecules, ammonia hydrolysis, hydrolysis and so on. For SIS, the most commonly used tube functional groups are amino and carboxyl groups [60] to bind to the carboxyl and amino groups of heparin, glycosaminoglycans or other targeting molecules.

Vascular disease is one of the diseases that endanger human health. The most fundamental treatment is vascular transplantation. But the source of autologous blood vessels is limited. Therefore, a large number of artificial blood vessels are clinically required as a transplant substitute. As early as 1989, the potential of SIS as an artificial blood vessel was investigated. Researchers implanted SIS as a large-diameter (10 mm) vascular graft into a dog’s renal aorta. They found that some dogs survived, but others died of embolism, arterial dilatation, adhesions and so on. In addition, the blood vessels of SIS materials are less flexible than natural arteries [52, 61]. Furthermore, when preparing a small-diameter (<6 mm) blood vessel, the blood flow velocity is low and the resistance is large, so thrombosis, aneurysms and obstruction are more likely emerge [62–64]. In order to improve the anticoagulant ability of small-caliber artificial blood vessels and improve the long-term patency rate, investigators from various countries have conducted extensive research on surface modification and endothelialization.

Thrombosis is the main factor affecting vascular patency and graft failure. Heparin is a well-known anticoagulant. Fixing heparin on the surface of the material can improve blood compatibility and reduce thrombus formation. There are many methods for immobilizing heparin on the surface of SIS, such as physical adsorption and functional group bonding. The physical adsorption binding force is too weak, and the quick release leads to a short-lasting effect [65]. Chemical integration is accomplished by ions and covalent bonds. The carboxyl group reacts with the amino group. Using a plasma generator to stimulate SIS, which will cause a large amount of aminocarboxyl radicals in a large amount of collagen and amino acids in SIS [66]. Soaking in heparin solution will form a large number of chemical bonds with heparin molecules on the surface of SIS to complete heparin fixation [67]. This is the application of plasma to the surface modification of biological materials. Moreover, by changing the parameters of the plasma, the physical and chemical properties of the biological material can be changed [68]. The amount of heparin bound to SIS can be determined by toluidine blue, and the activity and release kinetics of bound heparin can be accurately determined by ELISA [46]. Studies demonstrate that EDC, *N*-hydroxysuccinimide (NHS), 2-morpholinoethanesulfonic acid (MES) and other crosslinking agents are also used to heparin. EDC and NHS participate in and promote the combination of amino groups on SIS and heparin to form imide bonds, thereby immobilizing heparin on SIS [69]. Heparinized SIS showed a significantly different surface morphology compared with untreated SIS. The heparinized SIS surface was wrinkled and textured and evenly coated with microdots, which appeared to have a layer of heparin adhesion [70]. Some researchers have made the SIS of the compound heparin into a small blood vessel and transplanted it into the femoral artery of the dog. Both *in vivo* and *in vitro* experiments have shown that heparin modification is effective and can be effectively antithrombotic compared with blood vessels made of pure SIS [71].

As a natural collagen-based ECM, SIS has good biocompatibility and is often used to expand bladder and bladder reconstruction. Traditionally, the patient’s own colon [72], ileum or stomach has been tried, but some adverse reactions often occur. So, researchers tried to repair the bladder bioscaffold with natural nonimmunogenic pig SIS [73], and the treatment effect has improved a lot. Some researchers use SIS to repair the dog’s bladder and achieve initial success [74–76]. Based on this, the researchers have combined HA with the cationic surface of the poly(lactide-co-glycolide)-nanoparticles (PLGA-NPs) through a noncovalent electrostatic attractant, and diffracted light by Zeta PALS (Brookhaven Instruments, Holtsville, NY) showed that the loading efficiency was satisfactory. Then the HA-PLGA-NP solution was placed on the SIS mucosa test and incubated overnight to obtain NP-modified SIS. In this way, NPs are incorporated into the natural material SIS to improve the heterogeneity of the SIS and provide regeneration capacity. Experiments have confirmed that SIS scaffolds can support bladder epithelial cells and smooth muscles and can rebuild bladder cells. Compared with unmodified SIS, SIS scaffolds show better endothelial growth and function properly [77]. There are also studies using SIS for ureteral replacement [78]. On the basis of bladder reconstruction, there must be a lot of unknowns to explore.

In addition, some scholars use the functional group to cofix the SIS on the surface of the dopamine-coated polypropylene mesh for reconstruction and repair of the prolapsed pelvis [79]. For reconstruction and repair of the prolapsed pelvis, the good biocompatibility of SIS is combined with the strong mechanical properties of the polypropylene mesh. Specifically, the SIS is dissolved in a solution containing a crosslinking agent, such as EDC, MES, NHS, etc., and a sample of the polypropylene mesh coated with dopamine is soaked to complete the crosslinking course. The final SIS product was also implanted into the vaginal mucosa of rats and showed good adaptability [80].

### Protein adsorption

Protein adsorption plays an important role in surface modification. The biological activity and special excellent properties of proteins can be introduced into the reconstruction and modification of biological scaffold materials. Because the adsorption of proteins and other active macromolecules on materials can provide a good bridge and nutrient transport system for materials and cells, it plays a huge role in the *in vivo* response [81].

**Figure 4.**
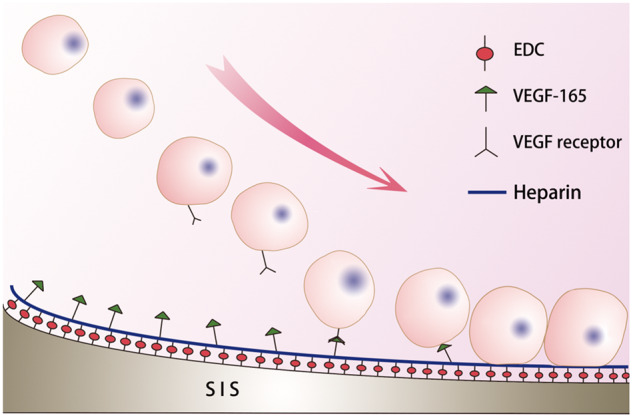
Schematic representation of heparin and heparin-bound VEGF-modified SIS-captured cells.

Koobatian *et al*. [46] have developed tissue engineered small blood vessels by SIS combining with heparin and VEGF. VEGF is a protein that induces angiogenesis *in vivo* [82]. The researchers sewed SIS piece into a tube of 4 cm in length and 4.5–4.75 mm in diameter. After crosslinking heparin with a crosslinking agent such as EDC, it was immersed in PBS containing VEGF for 8 h and then implanted into the carotid artery of the sheep. The activity of VEGF bound to SIS and the release kinetics of VEGF can be detected by ELISA [46]. Immobilization of VEGF on SIS led to high patency rates and complete endothelialization of the SIS lumen within 1-month post-implantation in the sheep [46]. With increasing surface concentration of VEGF, the number of attached endothelial cells (EC) was increased and reached a maximum between 700 and 1000 ng/cm^2^ of VEGF. Further, under a range of shear stresses from low (0.5 dyne/cm^2^) to physiological (15 dyne/cm^2)^ condition, the microfluidic device demonstrated that immobilized VEGF was highly selective for EC but no capture of other kinds of cells [82]. Figure 4 shows schematic representation of heparin and heparin-bound VEGF-modified SIS-captured cells. Three months later, the blood vessels were patency, there was significant host cell infiltration, there was a fusion endothelium and it was arranged along the direction of blood flow. Both mechanical properties and vasoconstriction are comparable with natural arteries.

At the same time, after binding large molecules such as heparin to the surface of SIS, the surface of SIS becomes rough, anticoagulation is enhanced, water contact angle is increased, hydrophilicity is enhanced and cell attachment is easier, thus obtaining higher endothelial coverage [83–85]. From this perspective, hydrophilicity is also a key factor on surface modification of SIS.

Some researchers have implanted bone morphogenetic protein 2-related peptide P28 (designed by the researcher) on the surface of SIS to prepare heparinized mSIS-heparin-P28 to guide osteoporotic bone regeneration and improve osteoporotic bone regeneration. A medical dilemma with low ability specifically the heparinized SIS is incubated in a solution of P28 peptide-containing bovine serum albumin. The experiment confirmed that after transplantation of mSIS-heparin-P28 into rats, it promoted cell proliferation, increased alkaline phosphatase activity and consistently enhanced expression of osteogenic-related genes. It is proved that mSIS-heparin-P28 after surface modification can significantly stimulate the regeneration of osteoporotic bone and also provides a good demonstration for the regeneration of bone and cartilage and tendon tissue [86].

### Mineral coating

Mineral coating technology has been in use since 1990 [87], and there are many applications for bioglass and ceramic for mineral coating and reconstruction of bone. Surface modification based on mineral coatings is mainly focused on the reconstruction and regeneration of bone and cartilage [36, 88]. The main point of bone tissue engineering is to find a suitable scaffold as a structural support and drug delivery carrier for tissue growth, which can produce and form bone cells and bone growth factors necessary for bone growth. Based on the good biological properties of SIS, some mineral ions are introduced to enhance the mechanical properties of the scaffold and change the overall properties of the scaffold material. The mineral particles are bound to the surface of the SIS scaffold from the ion-rich solution in the form of inorganic crystal particles for surface modification.

Researchers have also combined SIS with mesoporous bioactive glass (MBG) to improve mechanical and biological properties and stimulate bone and angiogenesis *in vitro* and *in vivo* [50]. Bioactive glass can uniquely combine with hard or soft tissues to stimulate new tissue growth and degradation but has the disadvantage of inherent brittleness and surface instability [89–91]. The researchers then mixed the MBG powder into the SIS solution at different ratios and poured it into a mold to freeze-dry it. After crosslinking in ethanol, the stent was obtained by freeze-drying. The surface of the scaffold has interconnected voids and a smooth interstitial surface. Both *in vitro* and *in vivo* experiments have demonstrated that the SIS-MBG scaffold exhibits angiogenic gene expression and tube formation ability and excellent mechanical and compressive properties. In summary, the SIS-BMG composite scaffold represents an exciting vascular and bone regeneration material.

Some researchers have dissolved SIS powder in water-based polyurethane (synthesized from polytetramethylene ether glycol, isophorone diisocyanate and 2,2-bis(hydroxymethyl)butyric acid) in order to make the polyurethane bind to the biologically active matrix of SIS to obtain a stereoscopic biological scaffold after chemical crosslinking. The hybrid scaffold showed high elasticity and high mechanical properties compared with the control group, and the Human Umbilical Vein Endothelial Cell (HUVEC) cells were excellent in the proliferation activity. Microvascular formation was also found subcutaneously in SD rats without inflammatory reaction [92]. Another study shows that bioceramic hydroxyapatite (Hap) is combined with SIS to construct a new porous scaffold for tissue engineering regeneration [93]. The SIS-Hap sponge scaffold has a distinct interconnected pore size similar to that of natural trabecular bone, thus providing an excellent bone tissue engineering scaffold to future generations.

### Topography and formatting modification

The surface modification of SIS is much more than above. Topography and formatting modification is also an important method of surface modification. Because topographic modification can alter the microenvironmental morphology of cell growth, it naturally also affects the activity of biological cells and the performance of biological scaffolds. Topographic modification is mainly to change the nanoscale environment that the adherent cells can recognize [94]. It mainly reduces the surface roughness of the material and increases the surface area and mechanical properties of the interaction, and hydrophilic. For example, some researchers have modified the nanobionic surface of SIS by plasma-initiated technology, making the surface of SIS rougher and the contact angle of water larger, i.e., enhanced hydrophilicity, enhanced anticoagulation and good endothelial coverage when transplanted into animals [25]. Some researchers have also designed an SIS bunk bed dressing [95]. Upper layer is a humid environment, whereas the lower layer is a low temperature gel layer, and the double layer has excellent mechanical properties, which is a promising wound accessory.

One method of topography and formatting modification is to reduce the roughness of the material. Because the single-layer barrier membrane is easily collapsed in tissue engineering repair, it affects repair and regeneration [96]. Therefore, some researchers have invented the SIS to stack to obtain multilayer SIS (mSIS). The outer surface of mSIS is smooth, and rough side is hidden into the inner laminate, which also improves the adverse effects of the rough surface of the SIS itself on repair [97]. NPs are often used to deliver drugs that bind to the SIS surface and increase the contact area for proliferation *in vivo* [98, 99]. PLGA is a common tissue engineering bioscaffold with good mechanical properties. One study demonstrates that combined HA-PLGA-NPs on the surface of SIS will maintain the surface porosity of SIS, increase the contact area and enhance the functionality of tissue regeneration [77]. PLGA-NPs can use the volume advantage to uniformly adhere to the surface of SIS, reduce the permeability of SIS to urea and enhance the proliferation of endothelial cells without changing the mechanical structure of SIS. Reduced urea permeability is particularly important for bladder function, as urine leakage represents a serious disorder of the bladder. Experiments have also confirmed a significant increase in neovascularization as well as bladder enlargement in the bladder enhancement model [77]. HA-PLGA-NPs are a novel nano method capable of modifying SIS while maintaining the porous structure of the SIS and providing a uniform structure.

Although SIS has been widely used in repair and replacement organizations, it must be made into a fiber shape if it is to replace connective tissue. Researchers have pointed out that biopolymers and biomaterials are difficult to form nanofibers such as alginates, chitosan and the like [100]. In order to solve this problem, the researchers mixed SIS with some synthetic polymers with good mechanical properties by electrospinning to make SIS nanofibers to meet actual needs. Electrospinning is a special form of electrostatic atomization of polymer fluids. At this time, the atomized and split material is a tiny jet of polymer and finally solidifies into fibers. Electrospinning can produce polymer filaments of nanometer diameter [101]. Based on this, researchers have dissolved and stirred SIS powder and poly(ε-caprolactone) (PCL) in a mixed solvent of dichloromethane and dimethylformamide and sprayed the fibers with an electrospinning machine. The receiving device can be a flat plate or a tubular glass rod. SIS can be embedded in PCL fiber well, and the surface of the received SIS presents filiform fibers, which meets the requirements for connective tissue reconstruction. Planting cells on the scaffold also showed that the cells were able to grow and proliferate normally. This discovery provides a good reference for the material modification of SIS. Nanofibers are increasingly being used in tissue engineering due to their high porosity and the ability to adjust performance through solution [102]. In addition, Kim *et al*. used SIS and PLGA to prepare hybrid nanofiber membranes by electrospinning to improve mechanical properties and compatibility [103].

In peripheral nerve construction, some researchers have incorporated SIS powder into PCL in different proportions, mixed and stirred to dissolve, and use electrospinning machine to prepare peripheral nerve conduit [104]. Results show that SIS has been successfully embedded in the PCL matrix, and the diameter of the PCL-SIS fiber is smaller than that of the pure PCL, which makes the inner and outer surfaces of the catheter denser, providing a better adherence and proliferation environment for the cells. PCL-SIS catheters also exhibit better surface water absorption and mechanical properties. On the basis of electrospinning, using low temperature 3D printing to prepare SIS into an SIS stent that can have a 3D porous structure with adjustable morphology and structure. The material and biochemical properties of the stent can be adjusted by changing the printing parameters [105].

### Drug combination

Effective healing of wounds requires a high degree of coordination of molecular biological events, including angiogenesis, epithelial formation and repair of the skin and tissues. These are the basis for the complete regeneration of tissues and organs by SIS as an *in vitro* biological scaffold. The drug-derived natural ECM can better reduce the toxic side effects of the drug and enhance the biocompatibility of the stent. Among the methods in which the surface of SIS is modified, there is another way to bind the drug on the surface of SIS to make the drug work.

For example, an anti-adhesion drug, nimesulide, was bound to the SIS surface by soaking, and animal experiments showed inhibition of postoperative adhesion in rats [106]. Some researchers have also used SIS of crosslinked methotrexate (Met) to treat rheumatoid arthritis (RA) by preparing a Met-loaded SIS injectable gel that is then injected into the joint to form a drug store [107]. The experiment showed significant RA repair and extensive regeneration. Mei *et al*. developed an Ic-ECM-SIS scaffold that uses ECM to deliver the drug icariin (LC) on the SIS scaffold by coating the SIS scaffold with LC drug [108]. The cells on the Ic-ECM-SIS scaffold can grow normally, and CD31 also has high expression, which has a certain promoting effect on angiogenesis. At the same time, the expression of some osteogenic genes, such as alkaline phosphatase and osteocalcin, is elevated. *In vivo* experiments showed that the rate of new bone formation was also higher than that of the control group, and no inflammatory reaction occurred [108, 109].

In addition to the success of artificial blood vessels made by SIS films in replacing the carotid arteries of the sheep, our team has narrowed the caliber of SIS blood vessels, opening a new dimension for the replacement of small-caliber blood vessels. We made a vascular sew into a small blood vessel with a diameter of 2 mm and a length of 2 cm, which was transplanted into a rabbit to replace the carotid artery [45]. In particular, we invented a small-caliber artificial blood vessel based on a two-layer SIS, using a mixture of curdlan curd and dipyridamole as a “sandwich” filler for the drug delivery system. Finally, we find that the patency rate of the double-layer SIS vessels was found to be higher than that of the control group, and the endothelialization was also higher than that of the control group. This demonstrates that the new hybrid small-caliber artificial blood vessel grafts show great improvements in vascular reconstruction.

## Conclusions

SIS can be easily obtained from the small intestine of the pig, and in the preparation process, the SIS can also be surface modified in various ways to adjust the mechanical properties and biological properties. It can be widely used in tissue engineering of various tissues and organs such as cartilage, heart muscle, bladder and skin. In order to summarize the five methods of surface modification of SIS, all the existing literature in this field were reviewed, and the goals and unique characteristics of each method were also discussed. Although each approach has a huge impact on the surface modification of SIS, all methods and their respective combinations deserve further exploration and application. The surface modification of SIS and the application of tissue engineering still have a long way to go. SIS is expected to be one of the ideal materials for repairing damaged tissues and organs in tissueengineering. Researchers should work to develop more modified methods for SIS to improve the performance of SIS to the extreme and comprehensive.

## Funding

This work was supported by the National Natural Science Foundation of China (No. 81571919) and LiaoNing Revitalization Talents Program (No.XLYC1907124).

*Conflict of interest statement*. None declared.
